# An experimental approach to study foraging memory in ectomycorrhizal mycelium

**DOI:** 10.1080/19420889.2025.2580130

**Published:** 2025-11-06

**Authors:** André Geremia Parise, Vinicius Henrique De Oliveira, Francesco Tamagnini, Mark Tibbett, Brian John Pickles

**Affiliations:** a School of Biological Sciences, University of Reading, Reading, UK; b School of Agriculture, Policy and Development, University of Reading, Reading, UK; c School of Pharmacy, University of Reading, Reading, UK

**Keywords:** Cognitive ecology, foraging, fungal behavior, fungal ecology, negative results

## Abstract

Behavioral ecology of fungi is an emerging field investigating how fungi respond to environmental stimuli through morphological and physiological changes. Progress requires methodologies suited to fungal biology. Here, we developed an experimental approach to test for memory in the ectomycorrhizal fungus *Laccaria bicolor*. We hypothesized that mycelium exposed to pea cotyledons would retain directional information about the nutrient source. To test this, a portion of the mycelium was transferred to fresh medium, where memory would be assessed by asymmetrical growth toward the former nutrient position. The hypothesis was not supported, but the methods offer a framework for exploring fungal behavior in both ectomycorrhizal and saprotrophic species. Although no evidence of memory was found, this study highlights the value of publishing both positive and negative results and provides tools to advance research on fungal cognition and behavior.

## Introduction

The behavioral ecology of fungi, also known as fungal ethology [1], is a recent research field that has gained traction in the last few years [2]. Such research has been carried out since at least the 1990 s (e.g., [3–5]), but it is only recently that it has emerged as a specific field. Studies on fungal behavior [6,7], memory [8], foraging, and decision-making [9] are now appearing in the scientific literature with more regularity. Some authors indeed proposed them as cognitive or even conscious [1,10]. Demonstrations of fungi with the abilities outlined above can also be glimpsed by other studies that did not focus specifically on these functions. For example, the decision-making process of the pathogenic fungus *Candida albicans* to switch from yeast to filamentous forms, includes the perception and integration of several cues from the environment such as temperature, O_2_, CO_2_, pH, serum, and signaling molecules from other cells to decide whether to continue as a yeast or switch to the hyphal form [11–14].

Despite these studies, gaps in the knowledge base surrounding fungal behavioral ecology remain vast, and our work aims to contribute to the field by investigating a phenomenon recently identified in fungi: memory. Memory can be described as the capacity to encode information about past experiences and recall them in the future, regardless of the system that manifests it [15,16]. One way of studying it is by observing how past experiences influence the actions of the system under study when the conditions that created the memory are no longer present or appear again after some time. Memory is the basis of learning, an important adaptive phenomenon that optimizes the interactions of the organism with the environment over time [15].

There is some evidence for memory in fungi. For example, *Saccharomyces cerevisiae* seems to store information of past events that helps it adapt to fluctuations of the environment in the future, which can be considered a form of memory and learning. Yeasts that had been submitted to hyperosmotic stress decreased the activity of the stress-responsive STL1 promoter, reducing the stress response to a subsequent hyperosmotic event [17]. In another study, unsuccessful mating created a memory that, when yeasts were exposed to mating pheromones again, transiently prevented them from budding. However, if they did not reproduce sexually in a short stretch of time, they would resume the asexual reproduction through the formation of buds [18].

In filamentous fungi, it could be useful to retain memory of the location of nutrient sources so as to find them again after the hyphae are severed. This possibility was demonstrated by Fukasawa et al. [8] when studying the directional memory of *Phanerochaete velutina*. The authors observed that, if these saprotrophic fungi were allowed to forage on a fresh piece of wood as its nutrient source (bait), then have their inoculum (the wooden block from where the fungus was growing) removed from the experimental setup and placed in a new one, they would grow more hyphae in the direction of where the bait had previously been located. The authors, nonetheless, honestly discuss that their results could be criticized because the directional memory could be explained not only by the fungi encoding the information about the direction of the bait, but simply because there would be more propagules on the side of the inoculum that faced the wooden bait [8].

In this work, we took inspiration from Fukasawa et al. [8] to design an experiment to test directional memory in an ectomycorrhizal fungus, *Laccaria bicolor*, that would: 1) potentially prevent the problem of uneven propagules outlined by Fukasawa et al. [8], and 2) offer an easier way of testing direction memory in fungi. In our case, instead of using soil trays for observing fungal development, which limits the species that can be used and presents space and time constraints, we would make a similar experiment on potato-dextrose-agar (PDA) medium in Petri dishes. Doing these experiments in Petri dishes has the advantage of being easier and cheaper to carry out, it requires less space and allows a higher number of experimental replicates.

Furthermore, to our knowledge, this is the first study of this kind using ectomycorrhizal fungi. Memory could be an important ability even to ectomycorrhizal fungi because although they obtain their carbon (C) from their host plant, they still need to uptake nutrients and water from the environment, so the ability to regrow hyphae toward sources of nutrients remains as important as it is to saprotrophic fungi to acquire C. Since the functional mutualism of the mycorrhizal system depends on a compatible exchange of solutes between both partners, there would be a selective pressure for fungal nutrient acquisition and consequent mycelial foraging behaviors.

We thus tested whether *L. bicolor* could recall the presence and direction of a past source of nutrients and grow more mycelia toward it. We hypothesized that: 1) *L. bicolor* would encode the direction of a discrete source of organic nutrients and grow more mycelium in that direction after part of the primed mycelium was transferred to a new medium; and 2) this effect would be more pronounced with fungi growing in a nutrient-depleted medium. The experimental set-up we developed aimed to solve the potential problem of more propagules on one side of the inoculum causing a growth bias that does not relate to memory [8], enabling unequivocal assessment of directional memory in a fungus.

## Material and methods

### ‘Priming’ of the fungi

**Figure 1.**
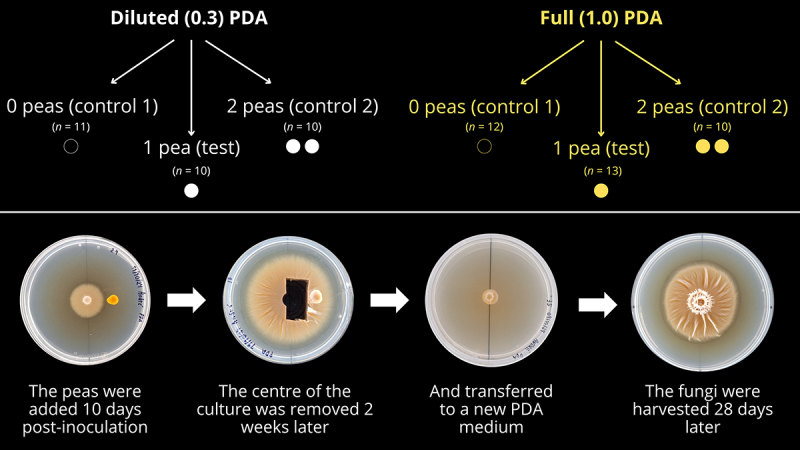
Diagram showing the experimental design above, with three treatments (Control 1, Test, and Control 2) for each condition (Diluted and Full PDA). Below, a step-by step guide of the procedure adopted, regardless of the number of peas.

A step-by-step diagram of the experiment is shown in Figure 1. Potato-dextrose-agar (PDA; Thermo Fischer Scientific, Waltham, Massachusetts, USA. Lot: 3,794,083) media were prepared in two concentrations: full concentration (39 g PDA · L^−1^), or at ⅓ of full concentration (13 g PDA · L^−1^) with added 10 g · L^−1^ of non-nutritious agar powder (Alfa Aesar-Termo Fisher Scientific, Heysham, UK. Lot: 10,231,469) for keeping the same consistency as full concentration. These were used for making, respectively, the two experimental conditions: full PDA (Full condition) and diluted PDA (Diluted condition). 25 mL of the media was poured onto standard acrylic 9 cm Petri dishes. Then, with a 0.5 cm-wide cork borer, agar plugs were removed from the growing edge of 50-days old *Laccaria bicolor* (strain S238N Maire P.D. Orton, originally provided by Francis Martin, INRA, Nancy, France, through Rodica Pena) kept in a fridge at 4 °C, and inoculated at the centre of the Petri dishes, which were sealed with Parafilm® (Bemis/Amcor, Zurich, Switzerland) and placed on the top shelf of an incubator (model INCU-270C, SciQuip, Rotherham, UK), internal dimensions (W x D x H): 60 × 60 x 75 cm. The top shelf was 25 cm below the ceiling panel of the incubator. The Petri dishes were kept in darkness at 18 °C. On alternate days, a line was drawn around the edges of the growing colony at the bottom of the Petri dishes, and then the dishes were randomly reshuffled to avoid any influence of the incubator on the direction of hyphal growth.

Ten days after inoculation, when the cultures growing in full PDA had a diameter of approximately 2.9 ± 0.1 cm (*n* = 37) and the ones growing in diluted PDA had a diameter of approx. 3.1 ± 0.2 cm (*n* = 34), the Petri dishes were randomly assigned to different treatments. The treatments consisted of: Test, where one yellow pea (*Pisum sativum*) cotyledon (hereafter, just ‘pea’) was placed 1.5 cm away from the center of the Petri dish; Control 1, where no pea was included in the Petri dish, and Control 2, where two cotyledons were placed equidistantly 1.5 cm from the center of the Petri dish in opposite sides. The yellow split peas (Lot: 82,670–1-1–1, produced in the UK; ASDA, Leeds, UK) were oven-dried at 40 °C until constant weight was achieved, then weighed in an analytical balance (Mettler AE160, Mettler-Toledo, Leicester, UK). Only peas that weighed exactly between 100 and 105 mg were used in this experiment. This is to minimize any effect of different mass in the growth direction of hyphae.

The peas were previously autoclaved in an open glass Petri dish for 5 minutes at 121 °C – counted from the moment the pressure indicator valve lifted – in a portable steam sterilizer (Classic, model 210048, Prestige Medical, Blackburn, UK). After this time, the sterilizer was turned off and a fan placed behind it to cool it as quickly as possible.

In a sterile laminar flow cabinet, the Petri dishes were opened, the peas were added, and then the Petri dishes were resealed with Parafilm®. Petri dishes for Control 1 (0 peas) were opened and subsequently closed again. All Petri dishes were taken back to the same incubator as before, in the same conditions, and incubated for 7 days, being randomly repositioned at alternate days.

### Transfer to a new medium

14 days after including the peas, all the Petri dishes were taken to the laminar flow cabinet again. They were opened and, with a sterile 1.0 cm-wide (internal measurement) cork borer, an agar plug was bored around the 0.5 cm plug that inoculated the plate. Then, sterile pieces of aluminium foil were placed on the edges of the plug where it intersects the line drawn under the Petri dish to mark the position of the plug. The plug was carefully removed with a scalpel and placed on the center of a new Petri dish with 25 mL of PDA at the corresponding dilution of the treatment (Full or Diluted) and in the same position as they were in the previous Petri dish, but without any peas present. These new Petri dishes had a line drawn at the bottom dividing it in two halves. With this arrangement, hyphae would have to grow down to the bottom of the plug towards the new agar before they started spreading radially, thus minimizing any propagule effect.

The new dishes were sealed with Parafilm® and taken to the same incubator as before, under the same conditions. After a few days, we noticed that the Petri dishes in the diluted PDA condition were contaminated due to a problem with the autoclave, but it did not seem to have affected the growth of the fungi. They just engulfed the bacterial colonies as if they were not there.

The Petri dishes were left undisturbed for 5 days in the incubator to allow the hyphae to penetrate the new agar from the plug, securing it in place. This was indicated by hyphae growing around the plug on the new agar. The Petri dishes were then removed from the incubator, a line was drawn around the edges of the colony at the bottom of the plate, and they were reshuffled before being taken back to the incubator. Drawing the line was always made by the same experimenter, holding the plate c. 30 cm away from the face and wearing an eye patch over the non-dominant eye to avoid distortions in the drawing due to parallax. This ensured the lines to be exactly above the edges of the colony.

### Harvest of fungi

28 days after transferring the center of the cultures to the new Petri dishes, the cultures were photographed with a Samsung Galaxy A54 cell phone (Samsung, Suwon, South Korea) with 50 MP resolution, following Rodrigues et al. [19] protocol for photographing microbial cultures (using an 11.5 cm-high observing tube instead of 23 cm, see [19]). Then, the fungi were stored in a cold room at 4 °C. They were removed one by one from the fridge over the next three days for collecting the biomass. For doing this, we modified the protocol of Karaduman et al. [20] and De Oliveira and Tibbett [21]. The PDA was removed from the Petri dish and placed in a larger, glass Petri dish with milli-Q water. Then, the fungi were microwaved in a Russell Hobbs microwave (model RHM2087B-TS, Failsworth, UK) at medium high power for c. 07:40 ± 2 min for diluted PDA plates, and 05:50 ± 01 min for full PDA plates. This was enough to lightly boil the water, effectively dissolving the agar underneath it. The difference in time between the conditions is because we noticed that *L. bicolor* growing on diluted PDA typically grew more hyphae into the agar, requiring more time to properly melt. The mycelium was then removed from the water, bathed in cold milli-Q water for a few seconds, then blotted dry on a paper towel. All the mycelia were dried in a drying oven at 40 °C until constant weight. Their masses were measured with the same Mettler AE160 scale mentioned above. The empty Petri dishes were photographed to show all the lines drawn. The empty Petri dishes with the concentric lines were photographed in the same way described above for analysis.

### Analyses

#### Growth and asymmetry

To measure growth rate, we used ImageJ (version 1.54, National Institutes of Health, Bethesda, Maryland, USA) to calculate the area of the mycelium that was on each side of the Petri dish using the polygon tool. The area of the whole mycelium was calculated by adding the area of both sides of the culture. We used a normalized index of asymmetry to check the position of the agar plug in relation to the reference line that divided the halves of the Petri dish (Eq. 1).

(1) $$A_{i}=\frac{A-B}{A+B}$$

Where $A_{i}$ is the asymmetry index, A is the area of the plug in one half of the Petri dish, and B is the area in the other half. If the $A_{i}$ of the plug was <−0.05 or >0.05, we recalculated the centre of the plug with ImageJ, and only then measured the area of the mycelium in both sides of the Petri dish. We used the same equation to calculate the asymmetry of the culture every day, for the 12 days of measurements. The value of $A_{i}$ can range from −1, which would indicate 100% of mycelium growth in the side B of the Petri dish (away from the pea, in the case of the test treatment), and 1, where all mycelium would have grown in the side A of the treatment (towards the pea in the case of the test treatment). $A_{i}=0$ indicates perfect symmetry of the culture, but we considered only the asymmetry indexes beyond the <−0.05 and >0.05 range as significant.

#### Morphology

During the experiment, we noticed that several *L. bicolor*, in particular those exposed to the peas, regardless of the number, assumed a distinctive morphology, forming ridges that radiated from the center of the culture. We used this as a parameter to analyze the effect of the peas on the fungi. With ImageJ, we used the *Circle Tool* to crop the culture. Then, prior to the analysis, we used the *Circle Tool* to remove the center of the agar plug from the image. The agar plug was above the plane of the mycelium and could interfere with the colour threshold due to the bright white fungal structures it had. To count the number of ridges, we used the command *Image > Adjust > Colour Threshold*. In the threshold adjustment window, we adjusted the ‘brightness’ histogram by placing the cursor at the slope on the brighter side (since the ridges appeared brighter in the photos). Using the *Magic Wand Tool*, we selected all the visible ridges. We manually checked for and removed any false positives before counting the ridges and measuring the area of the image covered by each ridge.

#### Nutrient analyses

To understand whether the results observed were due to fungi exposed to peas being better nourished than the ones not exposed to peas, we analyzed the content of nutrients in the mycelia as follows:

##### Nitrogen and carbon determination

We quantified nitrogen (N) and carbon (C) in the mycelia using elemental combustion analysis. Due to the small dry mass of each sample, we pooled at least three mycelia to form one sample, resulting in three samples per treatment per condition. Mycelia from at least three replicates were ground with a pestle and mortar in liquid N_2_, then dried at 70 °C for four days. We used 100 mg of ground mycelium to determine C and N content with a Leco CNH 628 analyzer (LECO Corporation, St. Joseph, MI, USA).

##### Mineral nutrients determination

For the mineral nutrient determination, we digested 50 mg of ground dried mycelium as described above with 6 mL of a HNO_3_ (69%) + 2 mL H_2_O_2_ solution (3:1 v/v), using an Ethos Easy 44-Max Microwave Digestor (Milestone Srl., Sorisole, Italy), dried plant tissue programme (heat up to 200 °C in 25 minutes and hold at 200 °C for 15 minutes). Samples were pre-digested in room temperature for 15 minutes before heating. Extracts were then filtered using Whatman 540 (Cytiva, Danaher Corporation, Wilmington, DE, USA) paper filter and diluted with ultra-pure water (UPW) to 50 mL. An aliquot of 2.5 mL was further diluted with 7.5 mL UWP (1:3 v/v) before analysis through inductively coupled plasma optician emission spectroscopy (ICP-OES) (PerkinElmer Avio500, PerkinElmer, Inc., Shelton, Connecticut, USA). Blank samples and the plant certified reference material (IAEA-359 cabbage leaves) were included for quality control. Elements determined were calcium (Ca), copper (Cu), iron (Fe), potassium (K), magnesium (Mg), manganese (Mn), phosphorus (P), sulfur (S), and zinc (Zn).

#### Statistical analysis

Data was analyzed by one-way ANOVA, followed by Tukey test to discriminate differences between each treatment (*p* < .05). Homoscedasticity was determined by the Levene test (*p* > .05), and normality by Shapiro-Wilk (*p* > .05). Dry mass data was transformed by $\mathrm{lo}g_{\left(x\right)}$ to attain normality. For other non-normally distributed data, Kruskal-Wallis tests (*p* < .05) were applied. All analyses were carried out using the software XLStat® (version 2019.2.2, Lumivero, Denver, CO, USA).

### Control for agar-borne chemicals

During the course of the experiment, we considered the possibility that substances like nutrients or hormones could be leaking from the peas and impregnating the agar which could then potentially be transferred with the fungal plugs depending on their spatial distribution. If true, the presence of these agar-borne chemicals might be the cause of any differences in morphology in the fungi previously exposed to the peas, rather than any internal mechanism for storing information. We also noticed that *L. bicolor* growing in full PDA detached very easily from the medium in comparison to that in the diluted medium. Hence, we carried out an additional experiment to control for the potential presence of chemicals exuded from the peas into the agar. With the exception of the nutrient and asymmetry analyses, the same experiment described above was repeated but, instead of transferring a 1 cm-wide agar plug as described previously, we only transferred to the new medium the disk of mycelium that was was on top of the agar plug, thereby controlling for any inadvertent transfer of agar-borne chemicals.

## Results

In this study, we tested fungi in two conditions: PDA with ⅓ of the original concentration (Diluted) and PDA with the normal, full concentration (Full). For each condition we used three treatments: Control 1 (no peas), Test (one pea), and Control 2 (two peas), with the hypothesis that the fungi in the Test treatment would grow asymmetrically towards where the pea was. Therefore, it could be argued that this would be an effect of the memory of the past presence of the peas in the medium, and not a simple physiological response to the presence of nutrients.

**Figure 2.**
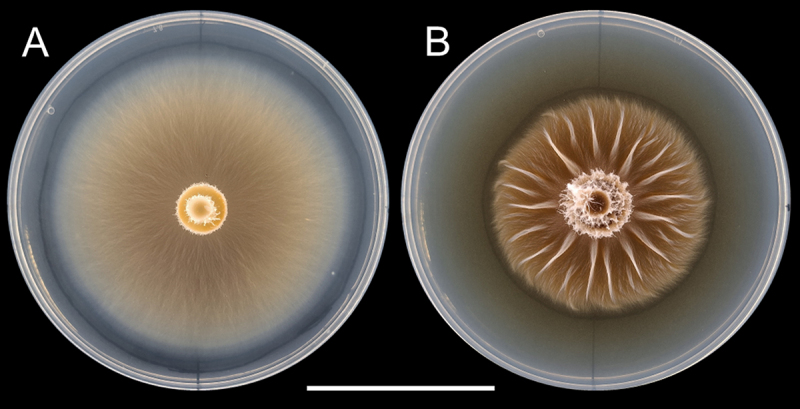
Different morphologies caused by the dilution of the PDA medium to *L. bicolor*. A: fungi growing in PDA medium at ⅓ of the original concentration. B: a fungus growing on full PDA. Note the smaller area and presence of thickened ripples as radial ridges in B compared to the diffuse growth pattern in A. The scale bar represents 4 cm.

Overall, the fungi from the Diluted condition grew more in area, regardless of the treatment, than the fungi in the Full condition. There was no significant difference in any growth parameter between the treatments within each condition (Table 1). Fungi in the Diluted treatment seem to have grown more mycelia inside the agar than those in the Full treatment, which rendered them much more difficult to remove from the agar than those in the Full agar. When microwaving them, it was impossible to separate all the agar from the mycelium and they kept a ‘slimy’ texture in the mycelium surface that had contact with the agar. Therefore, the data regarding the dry mass, C and N proportions, and the mineral composition of this group is unreliable and cannot be compared to the Full group. Additionally, fungi grown in the Diluted condition were paler and smoother when compared to the ones grown on Full condition, which were heavily ornamented (Figure 2).

**Table 1.** Average area and dry mass of the fungi in each condition and treatment (indicated as number of peas in previous Petri dish), with standard deviation. Fungi in the Diluted condition grew more in area than those on Full PDA, and there was no significant difference in the growth parameters between the treatments. *n* = number of replicates. 0 peas = Control 1; 1 pea = test; 2 peas = Control 2. Different letters represent significant differences between treatments after one-way Anova, followed by Tukey test (*p* < .05).

Condition	*n*	Peas	Area (cm^2^)	Dry mass (mg)
Diluted	11	0	44.7 ± 4.6 a	114 ± 24 a
	10	1	44.2 ± 3.9 a	111 ± 18 a
	10	2	42.4 ± 2.8 a	115 ± 24 a
Full	12	0	27.4 ± 8.4 b	87 ± 35 b
	13	1	23.7 ± 4.3 b	79 ± 21 b
	11	2	29.4 ± 8.3 b	104 ± 36 b

The presence of one or two peas did not have any significant effect in the concentration of C, N, and several mineral nutrients (Table 2). The dry mass, C and N percentage, and the mineral content were essentially the same across all the treatments.

**Table 2.** Percentage of total C and N in the mycelia, and concentration of mineral nutrients. For each treatment, three mycelia were ground together. Diluted = fungi grown on PDA diluted at ⅓ of the original concentration. Full = fungi grown on PDA at normal, full concentration. 0P = Control 1; 1P = test, 2P = Control 2. There were no significant differences among pea treatments after one-way ANOVA, except for K in the Diluted condition (in bold), where different letters correspond to significant differences after Tukey test (*p* < .05).

Condition	Peas	C %	N %	Ca (mg g^−1^)	Cu (µg g^−1^)	Fe (µg g^−1^)	K (mg g^−1^)	Mg (mg g^−1^)	Mn (µg g^−1^)	P (mg g^−1^)	S (mg g^−1^)	Zn (µg g^−1^)
Diluted	0	46.9 ± 0.4	2.0 ± 0.2	0.50 ± 0.04	5.5 ± 1.6	40.3 ± 14.5	**1.6 ± 0.1 ab**	0.40 ± 0.03	3.1 ± 0.3	5.2 ± 0.3	3.0 ± 0.1	12.0 ± 0.6
	1	47.0 ± 0.3	2.0 ± 0.2	0.48 ± 0.03	4.5 ± 0.3	34.5 ± 7.6	**1.3 ± 0.2 b**	0.39 ± 0.06	2.9 ± 0.6	5.2 ± 0.9	2.8 ± 0.1	13.2 ± 0.6
	2	47.0 ± 0.5	2.0 ± 0.3	0.48 ± 0.1	10.7 ± 9.3	26.0 ± 5.9	**1.9 ± 0.2 a**	0.43 ± 0.06	2.9 ± 0.6	5.4 ± 1.3	2.9 ± 0.1	17.1 ± 8.3
Full	0	49.9 ± 0.3	4.4 ± 0.1	0.36 ± 0.1	6.9 ± 0.5	71.0 ± 18.8	0.50 ± 0.1	0.20 ± 0.04	4.5 ± 0.5	4.3 ± 0.2	2.3 ± 0.1	43.6 ± 2.5
	1	50.2 ± 0.2	4.7 ± 0.1	0.44 ± 0.1	8.2 ± 1.0	73.0 ± 10.2	0.72 ± 0.1	0.28 ± 0.03	5.6 ± 0.5	5.4 ± 0.3	2.9 ± 0.2	54.0 ± 4.8
	2	49.8 ± 0.3	4.1 ± 0.1	0.45 ± 0.1	10.7 ± 4.5	91.9 ± 48.0	0.56 ± 0.1	0.19 ± 0.02	4.2 ± 0.3	4.1 ± 0.2	2.5 ± 0.2	45.5 ± 2.6

The asymmetry analysis did not show any growth preference for the side where the peas were in any of the days analyzed, as shown in Table 3. The fungi grew consistently in a circular shape. This effect was observed even in the fungi before transfer, when the peas were still present, as confirmed by Kruskal-Wallis (*p* > .05).

**Table 3.** Asymmetry index for the fungal cultures at each day of measurement. Positive values indicate more mycelium towards were the pea was, and negative values, more mycelium away from the pea. We considered values between −0.05 and 0.05 as indicating perfect symmetry, i.e., no growth preference for any side. There was no significant difference between the treatments in each condition after one-way ANOVA (*p* > .05).

Condition	*n*	Peas	Days after transfer												
			0	5	7	9	11	13	15	17	19	21	23	25	27
Diluted	11	0	−0.01	−0.01	−0.02	−0.01	0.00	0.01	0.01	0.00	0.00	0.00	0.00	0.00	0.00
	10	1	−0.01	−0.03	−0.02	−0.02	0.00	0.00	0.00	0.00	0.00	0.00	0.00	0.00	0.00
	10	2	0.00	−0.01	−0.02	−0.01	0.00	0.00	−0.01	0.00	0.00	0.00	0.00	0.00	0.00
Full	12	0	0.00	0.00	0.00	0.01	−0.01	0.00	0.01	0.00	0.00	0.01	0.00	0.03	0.01
	13	1	0.02	0.00	0.01	0.01	0.02	0.02	0.02	0.01	0.01	0.01	0.01	0.01	0.00
	11	2	0.00	−0.01	0.00	−0.01	−0.01	0.01	0.00	−0.01	−0.01	0.00	0.00	−0.01	0.00

**Figure 3.**
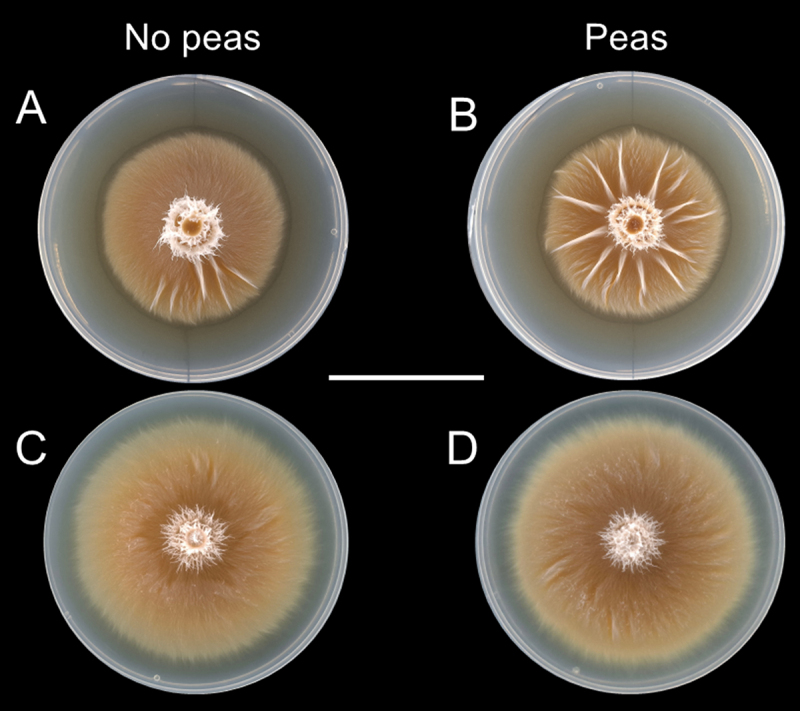
Expression of growth variation of *Laccaria bicolor* cultures grown on agar with and without peas, and in the presence or absence of retained agar. Left: fungi previously not exposed to the peas. Right: fungi exposed to the peas. In the Full PDA condition, when the mycelium was transferred with the agar plug to new PDA, the fungi previously not exposed to the peas developed significantly less ridges (A) than those previously exposed to them, regardless of the number of peas (B). When the mycelium was transferred without the agar plug, this effect disappeared, and there was no significant morphological difference between the fungi not exposed to the peas (C) and those exposed to them (D). The scale bar represents 4 cm.

In the first test, we noticed that the fungi exposed to the peas, regardless of the number, assumed a different morphology than the fungi not exposed to them. They present a significantly higher number of radial ridges that departed roughly from the center of the culture (Figures 2b, 3a,b). When we controlled for agar-borne substances derived from the peas by transferring only the mycelium without the agar plug with them, this effect disappeared, and their area was significantly larger (Figure 3, Table 4).

**Table 4.** Number of ridges and area of ridges in experiment 1 (fungi transferred with agar plug) and subsequent experiment 2 (fungi transferred without agar plug). Experiment 1 consisted of 3 treatments with different numbers of peas, and experiment 2 only had 2 treatments (0 and 2 peas). Different letters correspond to significant differences within each experiment, after one-way ANOVA and Tukey test (*p* < .05).

Experiment	*n*	Peas	*n*ridges		Area ridges
1	12	0	6.7 ± 6.2 b	0.07 ± 0.05 a	
	13	1	13.0 ± 5.3 a	0.07 ± 0.03 a	
	10	2	13.5 ± 6.1 a	0.09 ± 0.02 a	
2	30	0	1.8 ± 2.7 A	0.04 ± 0.04 A	
	22	2	2.7 ± 3.1 A	0.04 ± 0.04 A	

All the raw data for these analyses (dry mass, N% and C%, mineral nutrients, area and asymmetry of the mycelia, and pictures) are available in the Supplementary Material.

## Discussion

In this investigation, we developed a method to study the putative directional memory of fungal mycelium in agar plates instead of soil trays (cf. [8]). Studying the behavioral ecology of fungi in Petri dishes has the advantage of being technically easier, simpler, and quicker than in soil trays. Additionally, it can be performed in simple incubators without the need for any specialized facilities or appliances.

Inspired by Fukasawa et al. [8], we tested whether the ectomycorrhizal fungus *L. bicolor* would present a directional memory of the past presence of a pea cotyledon in its vicinity as a source of nutrients, particularly N and P. To the best of our knowledge, this is the first study to address memory ability of an ectomycorrhizal fungus. After incubating fungal cultures with none, one pea on one side of the culture, or two peas (one on each side), we transferred the center of the culture to a new Petri dish with the hypothesis that the fungi incubated with just one pea would asymmetrically grow mycelium preferentially towards the direction where it had contacted the pea in the previous petri dish.

Our first observation was that the fungi growing in diluted PDA grew over a greater area than the ones in the full concentration. They also seemed to attach more to the agar, which could suggest that the fungi in this condition were exploring for more nutrients. It was not possible to conclusively determine if they grew more or less dense mycelium – which would support this claim – because the attachment to the agar implied that some of the agar was embedded in the mycelium when we measured the dry mass. Statistical differences were detected between dry masses of Diluted and Full conditions, however, because of the higher agar attachment in the mycelia from the former condition, we used only area measurements to draw conclusions when the two media were compared.

Within each condition, we did not observe any significant change in the dry mass, C and N proportion, and mineral content of the mycelia across the different pea treatments. This may suggest that the peas did not have a significant nutritional effect on the fungi (which could explain the negative result) or that the effect was so small that it cannot be detected by these analyses. It is noteworthy that, although we could not measure this quantitatively in our experiment, we observed that the fungi seemed to have at least partly digested the peas. In a preliminary test, we noticed that the pea ‘dissolves’ almost completely after a few weeks under the mycelium (Supplementary Figure S1).

Regarding the main goal of this study, in none of the conditions (Full or Diluted PDA) did the fungi show any growth preference towards or away from the direction of the peas. We did not obtain an asymmetry index greater than 0.05 or smaller than −0.05 in any day of the measurement period, and towards the end of the test, this index was essentially 0.00 in all conditions and treatments. With this result, we can conclude that *L. bicolor* did not show any directional memory in this experiment.

The initial observation of significantly different morphologies (radial ridges) between fungi previously exposed to the peas compared to those not exposed was not found again when instead of transferring an agar plug with the mycelium, we transferred only the mycelium. Therefore, it raises the intriguing possibility of unidentified compounds leaching from the autoclaved peas, impregnating the agar, traveling over 1 cm in less than two weeks towards the center of the Petri dish, and staying there for several days, active enough in the transferred plug to induce the formation of radial ridges in the cultures and suppress growth in area.

We did not investigate which compounds these could be, but they would likely be plant hormones, conformationally resistant to autoclaving, that leached from the peas, such as auxins and cytokinins. It has been known for several decades that auxin can stay in agar for long enough to cause growth and morphological changes in plants [22], and the auxin indole-acetic acid (IAA) can remain stable after autoclaving at 120 °C for 20 minutes [23]. Similarly, the cytokinins trans-zeatin (tZ), 6-(γ,γ-dimethylallylamino) purine (2iP), kinetin, benzyladenine (BA), and *m*-topolin conserved their stability after autoclaving at 121 °C for 30 minutes [24]. Both cytokinins and auxins are present in pea seedling extracts [25] and are known to influence the physiology of ectomycorrhizal fungi [26–28]. In high quantities, IAA combined with cytokinins inhibited the growth of *Suillus variegatus* [26]. If they have a similar effect on *L. bicolor*, this could partly explain why the fungi exposed to peas grew over less area (Figure 2). It is interesting, though, that the effect of this unknown compound was only visible in the full PDA condition, revealing some kind of context-dependency in the fungal response to it. Future studies should try to identify which substance has such a strong effect on the structure of *L. bicolor* mycelium, as it may prove useful to deepen the understanding of the physiology of this ectomycorrhizal species and give insights into how to manipulate its growth in agricultural and forestry contexts.

In this study, despite promising initial observations, we could not corroborate our hypothesis. Fungi exposed to a single nutrient source did not grow in the direction of a previously contacted source following mycelial transfer to a new medium. One of the possible reasons for this is because *L. bicolor* is an ectomycorrhizal fungus and, in this case, it was growing in axenic conditions, i.e., in PDA medium without a plant host. Despite growing rather well and appearing healthy, the fact that it was not in symbiosis with a host could have an influence on how it interacts with the environment. For directional memory, if this happens at all, it could be hypothesized that the fungus uses the plant root/s to which it is attached, and from which it receives carbohydrates, as a reference point and navigates outwards from there. When in culture, it would assume the standard radial growth common to many fungi. Evidently, memory could have occurred at metabolic and epigenetic levels, but these were not addressed here, as our initial interest was on the concept of spatial memory and how this would affect fungal growth. Another alternative hypothesis is that the transfer to new medium and subsequent measurements was too stressful for the fungus, and it lost the memory of the pea positioning. However, we note that preferential growth towards the pea was not observed even when the pea was present in the medium before transfer.

Despite yielding null results in this case, we still believe this methodology can be fruitful for the study of the behavioral ecology of fungi. We have demonstrated that the center of an ectomycorrhizal mycelium growing on agar can be extracted, transferred to a new growth medium, and it continues to grow without difficulty. It would be worthwhile testing this same set-up with other species of ectomycorrhizal fungi and with saprotrophic fungi, to explore whether their response would be different from that of *L. bicolor*. Instead of peas, other, bespoke sources of nutrients could be used to control for hormones or other undesired substances that could affect the results. The study of fungal behavioral ecology is in its early stages, and the development of appropriate methodologies is essential. Although the outcome did not conform to our predictions, this work is another step in the direction of building a framework to study how fungi perceive and interact with the world.

## Supplementary Material

Supplemental Material

## Data Availability

All the raw data relevant for this research is available as supplementary material available at: Parise, André Geremia; De Oliveira, Vinicius Henrique; Tamagnini, Francesco; Tibbett, Mark; Pickles, Brian John (2025), “An experimental approach to study foraging memory in ectomycorrhizal mycelium – Supplementary Material,” Mendeley Data, V1, doi: 10.17632/phstb5yttk.1
