# Supplementary figures and images for: An experimental approach to study foraging memory in ectomycorrhizal mycelium
Source: Commun Integr Biol. 2025 Nov 6;18(1):2580130. doi: 10.1080/19420889.2025.2580130 (PMC12599362; doi:10.1080/19420889.2025.2580130)

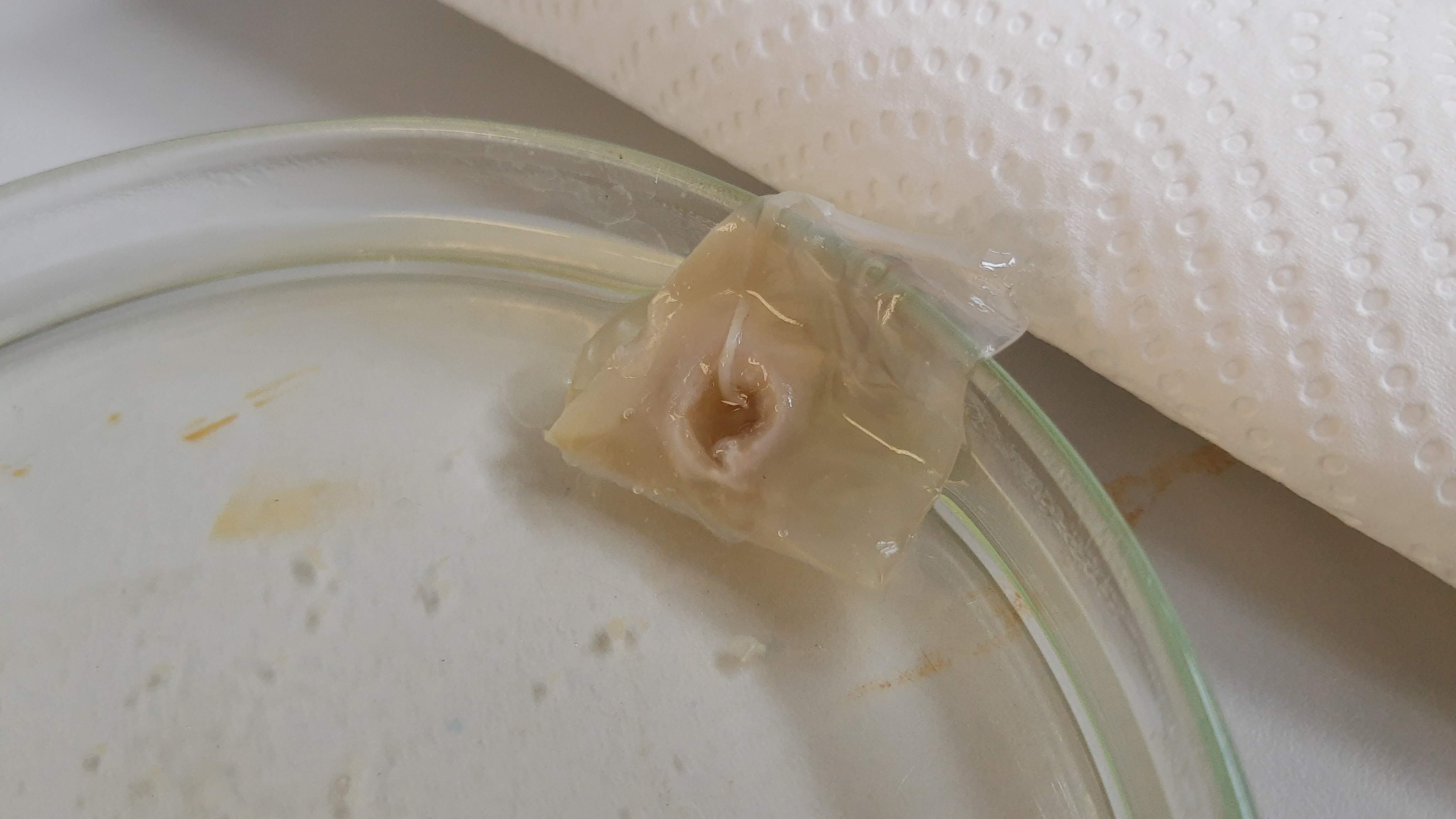

Supplement: Supplemental Material [file KCIB_A_2580130_SM9836.jpg]
